# A novel MPPT approach for photovoltaic system using Pelican optimization and high-gain DC–DC converter

**DOI:** 10.1038/s41598-025-24000-z

**Published:** 2025-11-18

**Authors:** Khadiza Akter, S. M. A. Motakabeer, A. H. M. Zahirul Alam, Siti Hajar Binti Yusoff, Rupendra Kumar Pachauri, Hasmat malik, Vinay Kumar Jadoun

**Affiliations:** 1 International Islamic University Malaysia, Jln Gombak, 53100 Kuala Lumpur, Malaysia; 2 Department of Electrical and Electronic Engineering, International University of Business Agriculture and Technology, Dhaka, 1230 Bangladesh; 3 Electrical Cluster, School of Advanced Engineering, UPES, Dehradun, Uttarakhand 248007 India; 4 Miyan Research Institute, International University of Business Agriculture and Technology, Dhaka, 1230 Bangladesh; 5 Department of Electrical Power Engineering, Faculty of Electrical Engineering, Universiti Teknologi Malaysia (UTM), Johor Bahru, 81310 Malaysia; 6 Department of Electrical Engineering, Graphic Era (Deemed to be University), Dehradun, 248002 India; 7 Department of Electrical and Electronics Engineering, Manipal Institute of Technology, Manipal Academy of Higher Education, Manipal, Karnataka 576104 India

**Keywords:** Maximum power point tracking, Photovoltaic system, Metaheuristics optimization, High gain converter, Pelican optimisation algorithm, Electrical and electronic engineering, Renewable energy

## Abstract

Photovoltaic (PV) solar cells are essential in renewable energy generation because they can produce power directly. As one of the most practical and widely used methods for meeting global clean energy demands, PV systems are integral to modern energy strategies. However, these systems face significant challenges in maximizing power output, especially under shading conditions and fluctuating loads. To overcome these issues, a, practical Maximum Power Point Tracking (MPPT) function is crucial for optimizing power extraction in such dynamic environments. Solar panels typically produce electrical outputs that vary in DC voltage, requiring a well-designed DC link interfacing circuit to ensure efficient energy transfer from the PV source to the load. In response to these needs, this study introduces the Pelican Optimization Algorithm (POA), a novel nature-inspired stochastic optimization technique designed to track the Maximum Power Point (MPP) of solar sources with high precision. This innovative MPPT method is coupled with a PV-fed, energy-efficient high-power DC-to-DC converter, which enhances MPPT operation by providing substantial step-up voltage gain and improved overall efficiency. An ideal PV model technique is employed in this study to accurately approximate the system’s mathematical parameters. The performance of the POA is benchmarked against three other Metaheuristics MPPT techniques: Particle Swarm Optimization (PSO), Harris Hawks Optimization (HHO),Gray Wolf Optimization (GWO), and Cuckoo Search (CS). These comparisons are conducted under uniform and partial shading conditions (PSCs) as well as varying load scenarios on a standalone PV system. The results reveal that the proposed MPPT technique excels in tracking the global maximum power point across diverse operating conditions. It offers rapid convergence, minimal MPP oscillation, quick response times (less than 0.2 s), and higher efficiency (99%). MATLAB/Simulink simulations further validate POA’s superior performance in MPP stability, tracking time, and effectiveness under PSCs.

## Introduction

Rapid technological development and population growth have created an insatiable need for energy supply, which is challenging to manage, as it has generated an insatiable energy demand that is difficult to meet using non-renewable energy sources. Sustainable renewable energy resources (RERs) are in demand due to the rapid depletion of existing energy supply systems and the detrimental effects of greenhouse gas emissions^1^. Despite being widely accessible, solar energy still faces challenges in terms of proper power generation and grid integration. The two main issues are (2) the rigid design of the already built conventional AC grid and (1) the installation expense and implementation difficulty of the Solar Photovoltaic System (SPV). Due to the high cost of implementation and poor ability to transform solar Energy into electricity, SPV has certain limitations. As a result, it is crucial to detect the maximum power from PV panels and transfer it to the load to maximize the effectiveness of solar photovoltaic (SPV) systems. PV panels reach their peak power at a specific operational point where the rate of power variation relative to voltage is zero. To track it, connect a compatible DC–DC converter to the PV panel, then use the maximum power point tracking (MPPT) method to operate the switch and match the impedance^2^. Additionally, for effective MPP monitoring, the input fluctuation of the DC–DC converter should be minimized.

Therefore, the MPPT algorithm and the DC–DC converter must be considered to enhance the overall efficiency of the PV source. In the literature, multiple MPPT techniques were discussed^3–5^. Due to the time-dependent behavior of PV systems under partial shadowing, the MPPT design for PV power systems should include features that track the global maximum power point (GMPP) under various conditions. To elevate the effectiveness of the solar system, a variety of MPPT approaches, including Hill Climbing (HC)^6^, incremental conductance^7–9^(IC), Perturb and Observe(P&O)^10,11^ have been developed. The P&O approach employs a perturbation in the solar system’s operational voltage, and the HC approach uses a disruption in the power converter’s duty ratio.

Due to the perturbation’s constant modifications required on either side of the PV curve to maintain the MPP, both approaches produce swings at MPP, resulting in power loss. A description of the two P&O algorithms that affect the factors of perturbation magnitude and perturbation rate is provided in^11^. The IC approach was proposed in^12^ to mitigate these fluctuations and increase module efficacy, thereby partially reduc Mitigate these fluctuations and increase A fuzzy logic controller (FLC) assists in tracking Ithe Maximum Power Point (MPP) in different weather conditions, such as irradiance and temperature changes, without the need to input system parameters. In^13^, an FLC and PI controller were combined to track the MPP while temperature and Iirradiance varied. While MPPT methods utilizing Artificial Neural Networks (ANN) can track the MPP under a variety of meteorological conditions well, these algorithms require regular tuning and are also complicated^14^. The cost of FLC- and ANN-based approaches is similarly high, and implementing them on hardware is challenging^15^. To increase the effectiveness, convergence level, and steady-state oscillation of MPP tracking under various weather circumstances, P&O and IC algorithms are combined with ANN and FLC. Because metaheuristic algorithms can regulate the nonlinear curve without using specific derivatives, they can track the global MPPT even in fluctuating weather conditions^16^. The literature describes a wide range of metaheuristic algorithms, including the genetic algorithm (GA), Cuckoo Search (CS), particle swarm optimization (PSO), bat optimization, and ant colony optimization^16–22^.

Selecting an appropriate DC–DC converter is ial for monitoring the Maximum Power Point (MPP), as iusingnes the input ripple factor and offers ,aase of input–output voltage ratios. DC–DC converters without utilizing transformers are recommended, as they provide a low step-up voltage that a conventional boost converter cannot produce^23^. They are fixed, and they were primarily because they are inexpensive and efficient. Voltage ratios can be stepped up and down, aiding the buck-boost converter. However, due to its discontinuous current supply, it cannot effectively track the Maximum Power Point (MPP). Despite having continuous input current and voltage regulation capabilities, SEPIC and Cuk converters feature huge ripples in input current that amplify the steady-state fluctuations near MPP and make tracking them challenging^15,24^.

A single-switch high-voltage gain converter is here selected as a solution to the problems discussed above, as it offers a wide variety of voltage conversion ratios and provides continuous current at the input with extremely low input ripple, which is helpful for effectively measuring the MPP. This effort aims to facilitate the Ingrid with the primary electrical grid and enhance the integration of the nanogrid with the primary electrical grid, thereby improving PV generation for such applications.The power transformation efficacy of the converter must also be sufficiently large to enhance the solar power tracking accuracy of the MPPT algorithm. The POA algorithm was created to increase tracking effectiveness under variable temperatures and Irradiance. An effective DC–DC converter has been presented to further improve the tracking and power conversion efficiency of the MPPT. As the converter is designed to slightly fluctuate the input current and efficiently supply harvested power to the load, it is connected to the PV panel to optimize maximum power point (MPP) tracking.

### Novelty of work

This section outlines the key innovations and contributions presented in the manuscript. Each of the following points reflects the original work and advancements made in the study, showcasing its significance in the field and setting it apart from existing literature.

- An emerging POA algorithm for MPP tracking offers fast convergence, minimal oscillation, moderate complexity, and firm performance in diverse weather conditions.
- Incorporation of a modified, extensible a high-gain, single-switch DC–DC converter with low input ripple and high efficiency.
- The algorithm is tested on a standalone system and can also be synchronized with grid applications.

In this research work, a novel approach to tracking maximum power using the Pelican Optimization Algorithm (POA) is implemented in conjunction with a power-quality DC–DC converter. This algorithm outperforms another existing algorithm in conjunction with the converter.

This paper is organized into seven sections. The introductory section includes a brief ooverview of the literature survey for MPPT, a high-level view of the literature survey for MPPT, and a high-level view of the literature survey for MPPT and high-performance converter topology. PV system modeling and specification are included in the second section. The operation and working of a new metaheuristic algorithm are stated in the third section. A brief analysis of the high-gain topology utilized as a solar interfacing circuit is discussed in Section Four. Sections present the simulation analysis, along with benchmarking and discussion. Concluding remarks are drawn in section seven.

## PV system modeling

**Fig. 1:**
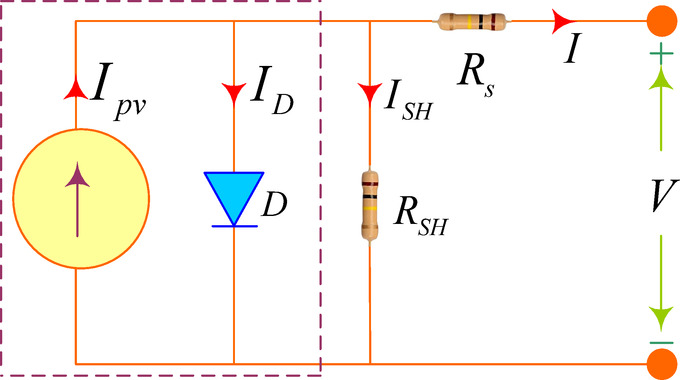
Single diode model of PV system.

To model a photovoltaic system, it is essential to use an accurate solar cell model. The photovoltaic system’s precise circuit, as shown in Fig. 1, is symbolized by a diode, a built-in resistance in series, and a shunted resistance. The inner series resistance (R_s_) of the PV signifies voltage loss, and the inner shunt resistance (R_SH_) represents leakage current. The two-diode device presents an excellent balance. This model performs better at e, requiring less computation time. This model performs better at low irradiances and takes less time to add. A photovoltaic system with Ns and Np modules coupled in a series–parallel (S–P) arrangement. The specifications of the solar panels used for this research are given below. Table 1 shows the photovoltaic specification of a single solar array used for MATLAB simulation to ensure uniform shading functionality. Applying KCL in Fig. 1 and Eq. (1) can be obtained.

1 $$I = I_{PV} - I_{D} - I_{RSH} \;{\text{and}}\;I_{D} = I_{S} \left( {e^{qV/nKT} - 1} \right)$$

where *I*_*S*_ is saturation current, *q* is charge, *n* is ideality factor, *K* is Boltzmann constant and *T* is temperature.

**Table 1:** Uniform shading parameters used for simulation.

Parameters	Specification
Series connected module per string	3
Number of parallel strings	2
Maximum power per module	349.59 W
Total maximum power	2095.54 W
V_MPP_	129 V
I_MPP_	16.26 A
Irradiance	100, 800, 600, 400, 200
Temperature	25°

**Fig. 2:**
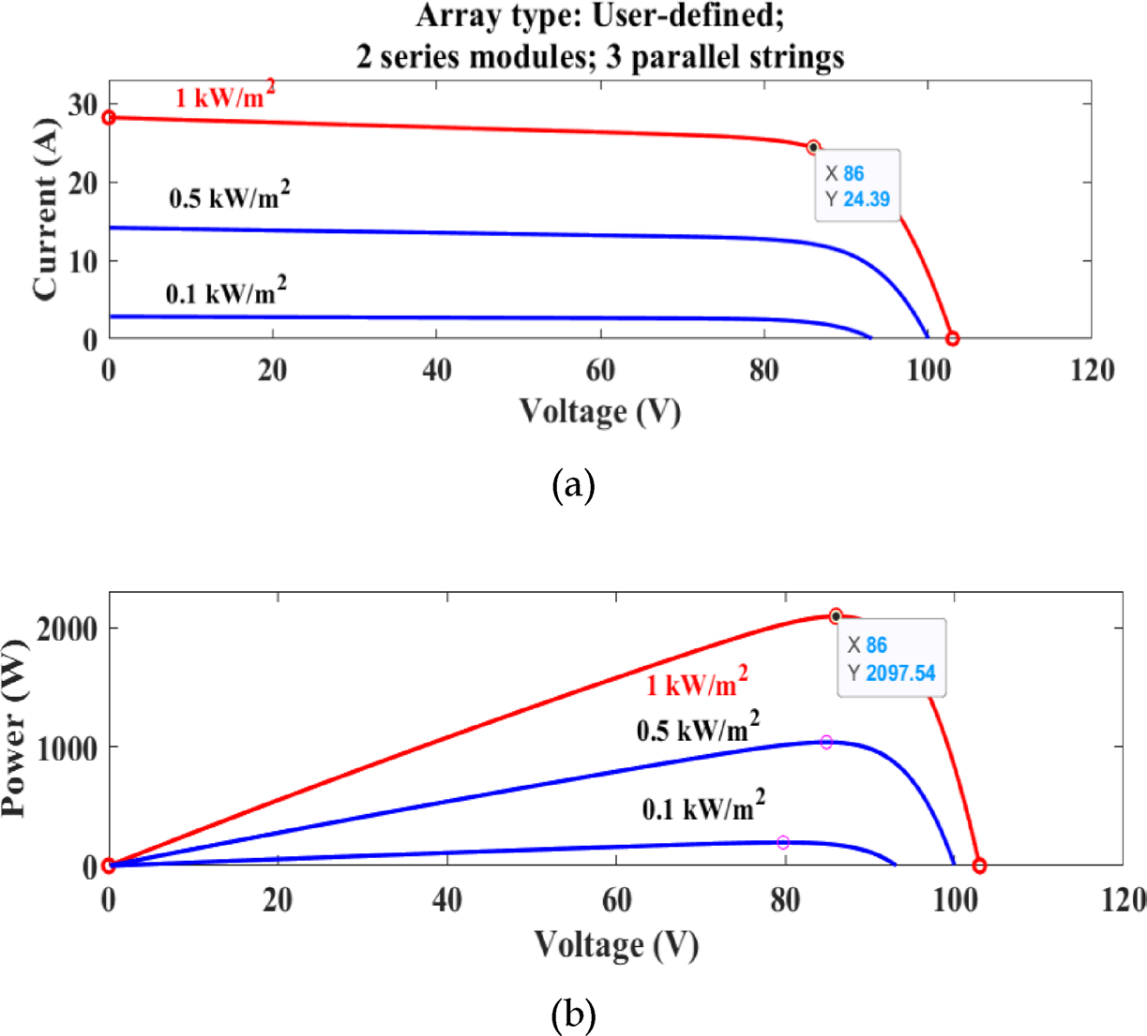
(**a**) P–V (**b**) I–V curves of solar PV array system.

The simulation output obtained after performing the simulation of the single solar array is given in Fig. 2. Table 2 shows the specification of a series-connected array to perform partial shading performance.

**Table 2:** Partial shading parameters used for simulation.

Parameters	Specifications
No. of PV array connected in series	4
Maximum power in each array	175 W
No. of cells in each module	72
V_OC_	43.99 V
I_SC_	5.17A
Temperature coefficient (%/deg.C)	− 0.3616
Temperature coefficient (%/deg.C)	0.041509
I_MPP_	4.78 A
V_MPP_	3 V
Irradiance level in 4 modules	650, 500, 200, 100 (W/m^2^)

**Fig. 3:**
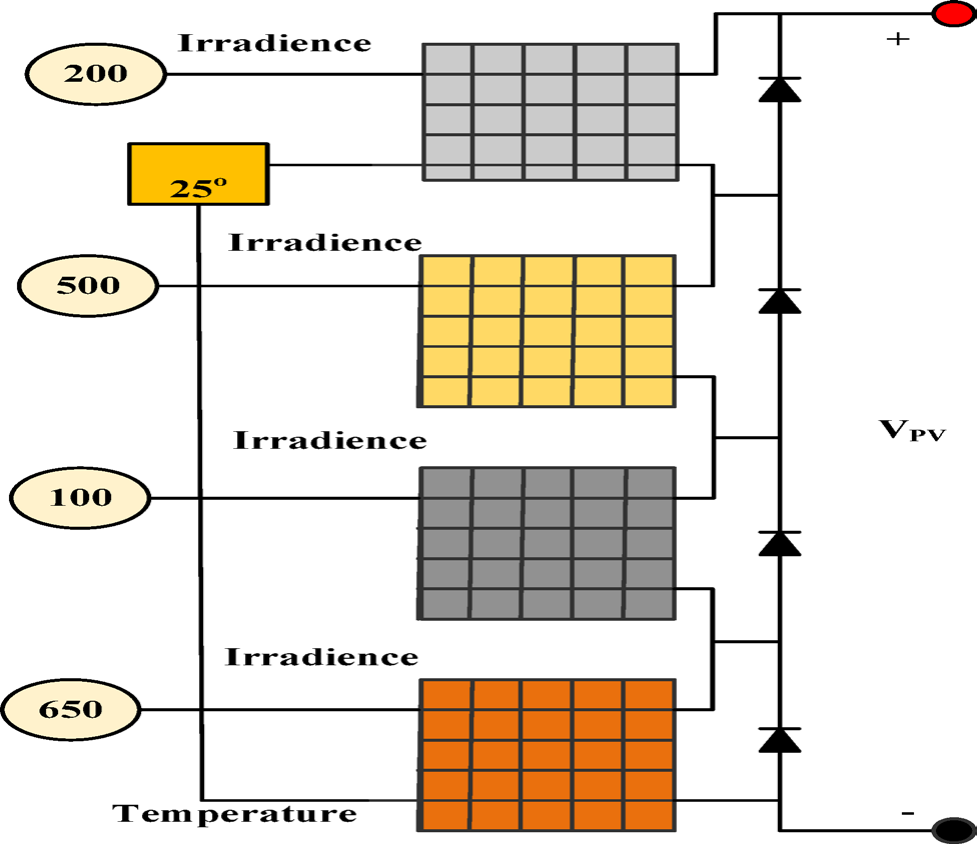
Series connected photovoltaic array for partial shading performance.

**Fig. 4:**
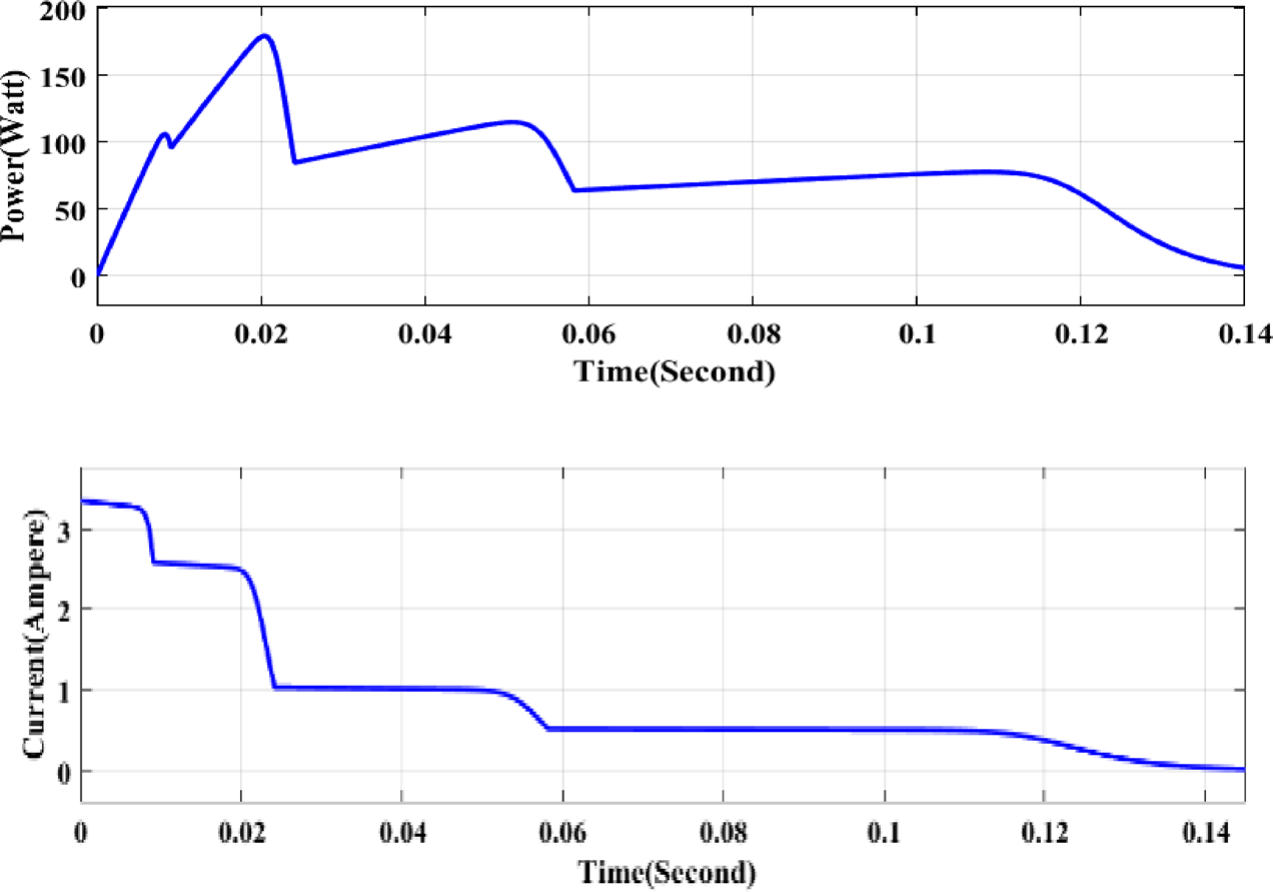
Power (**a**) and current (**b**) waveform of a partially shaded PV array.

Figure 3 demonstrates the Simulink model of several arrays used for partial shading simulation. Figure 4 shows the partial shading graphical characteristics in terms of power and current. Each array runs with different irradiance values, andfor this reason, several global and local maximum peaks are observed in Fig. 4a and b.

## Modelling of the Pelican optimization algorithm (POA) approach

The pelican uses an enormous pouch in its throat to catch and consume prey. It also has an extended beak. This species, which inhabits groups ofnumerous pelicans, loves socializing and living in large groups. Pelicans mostly eat fish, with turtles, frogs, and crustaceans appearing less frequently. When they are starving, they will even consume seafood. Pelicans often work together when hunting. The pelicans plunge from a height of 10–20 m to reach their meal after they have found it. Their expanding wings on the water’s surface push the fish to move to shallow water, where they can more easily catch fish. The pelican’s beak fills with water when it captures a fish. It moves its head forward before eating the fish to get rid of the extra water. The primary source of inspiration for developing the proposed POA was modeling the previously mentioned plan.

### Mathematical concept of proposed POA

The population-based Pelican optimization method ent that incorporates them as a component of its solution^25^. Determining where they are in the search space, each sample member recommends values for the variables in the optimization issue. Using Eq. (2), both the upper and lower bounds of the problem are used to initialize the population units arbitrarily.

2 $$x_{i,j} = Lb_{j} + {\text{rand}} * (Ub_{j} - Lb_{j} )$$

where I = search agent(pelican), Total number of search agent & D = design variable

$${\text{i}} = {1},{2},{3},{4} \ldots . \ldots \ldots \ldots .{\text{ N}}$$

$${\text{j}} = { 1},{2},{3},{4} \ldots \ldots \ldots \ldots ..{\text{m}}$$

$x_{i,j}$ = the jth variable’s value as shown by the ith possible solution. Rand is random, and Upper bound (U_b_) and lower bound (L_b_) are the respective symbols. The problem has m variables, where N is the total population.

The number of population members who reside within the proposed POA is identified using the population matrix. The objective function of the given problem can be assessed using each possible solution. A vector called the objective function vector is utilized to ascertain the values obtained for the objective function.

3 $$F = \left[ {\begin{array}{*{20}c} {F_{1} } \\ \vdots \\ {F_{i} } \\ \vdots \\ {F_{N} } \\ \end{array} } \right]_{{{\text{N}} \times {1}}} = \left[ {\begin{array}{*{20}c} {F_{(X1)} } \\ \vdots \\ {F_{(Xi)} } \\ \vdots \\ {F_{(XN)} } \\ \end{array} } \right]_{{{\text{N}} \times {1}}}$$

where F is the objective function vector and Fi is the objective function value of the ith candidate solution. The suggested POA modernizes possible solutions by modeling the behavior and strategies used by pelicans to attack and catch prey. This is a two-part hunting approach: the Exploration phase and the Exploitation Phase.

During the initial phase, the pelicans find their prey and dive towards it in the first step. The most crucial feature of POA is the random generation of the prey’s position within the search field. The pelican’s movement to its intended prey and the previously described concepts can be expressed numerically as follows:

4 $$x_{i,j}^{{{\text{phase}}_{1} }} = \left\{ {\begin{array}{*{20}l} {x_{i,j} + {\text{rand}}*(P_{j} - I*x_{i,j} ),} \hfill & {{\text{Fitness}}_{p} < {\text{Fitness}}_{i} } \hfill \\ {x_{i,j} + {\text{rand}}*(x_{i,j} - P_{j} ),} \hfill & {{\text{else}}} \hfill \\ \end{array} } \right.$$

where $x_{i,j}^{{{\text{phase}}_{1} }}$ is the new position based on phase 1 in the jth dimension *P*_*j*_ is the prey position, I is the random number in between 1 and 2. $x_{i,j}$ is the older position. The updated position for a pelican will be adopted in the proposed POA if the rate of the target function is increased in that state. Researchers named it as "effective updating." If the new fitness value is better than the older one, then we will update the value following the formula below.

5 $$x_{i} = \left\{ {\begin{array}{*{20}l} {x_{i}^{{P_{{1}} }} } \hfill & {{\text{F}}_{i}^{{P_{{1}} }} \angle {\text{F}}_{i} } \hfill \\ {x_{i} } \hfill & {{\text{else}}} \hfill \\ \end{array} } \right.$$

Here $x_{i}$ is the new updated position, the $x_{i}^{{P_{{1}} }}$ new position based on phase 1, $F_{i}^{{P_{1} }}$ is the fitness assessment according to phase 1.The pelicans reach the water’s surface in the next stage, stretch their wings to push the fish up, and scoop the meal into their neck pouch and this is the second portion of feeding. Thi process enhances the POA’s capacity for local search a The areas close to the pelican site require mathematical investigation to ensure the strategy converges to an improved outcome.

6 $$x_{i,j}^{{{\text{phase}}_{2} }} = \left\{ {x_{i,j} + R*\left( {1 - \frac{t}{T}} \right)*(2*{\text{rand}} - 1)*x_{i,j} } \right.$$

$x_{i,j}^{{phase_{2} }}$ new position of ith pelican based on phase 2 in the jth dimension. $x_{i,j}$ is the older position. R is constant whose value is 0.2 whereas t is the current iteration and T is the maximum iteration. The circumference of the neighborhood of the population elements to search individually near every individual to converge to an improved outcome is represented by the coefficient "R*(1 − t/T)". To get closer to the ideal global solution, this coefficient works well on the POA exploitation power. Because of the huge amount of this coefficient in the starting iterations, a greater area surrounding each member is taken into consideration. The "R*(1 − t/T)" coefficient falls as the method replicates more, leading to smaller radii for each member’s neighborhood. For the POA to come together to solutions that are nearer to the global (and even precisely global) optimal based on the usage notion, this enables us to scan the region surrounding all members of the population in smaller and more precise steps.

7 $$x_{i} = \left\{ {\begin{aligned}& {x_{i}^{{P_{2} }} } \hfill \quad {F_{i}^{{P_{2} }} < F_{i} } \hfill \\ &{x_{i} } \hfill \qquad {{\text{else}}} \hfill \\ \end{aligned} } \right.$$

$x_{i}^{{P_{2} }}$ new position based on Phase 2 and fitness value based on Phase 2 (Table 3).

**Table 3:** Summary of various algorithms highlighting key features.

References	MPPT techniques	P_m_(W)	PV system size	Improved GMPP (%)	Irradiance (W/m^2^)	Tracking time (s)
^9^	INC	100.17	1 × 1	1.811, 1.179, 1.615	1000–800	NA
^26^	Semi pilot cell FOCV	NA	NA	0.93, 11.01, 0.89 10.98, 0.83, 11.03	1000–200	NA
^27^	CSAM and modified FOCV	60	4 PV modules in series	7.27	1000–300	0.43, 0.49
^28^	FOCV via a special online process	245.328	3 PV modules in series	89.67, 0.51	1000–200	NA
^29^	FSCC algorithm	145	1PV module	13.33	NA	0.7
^30^	FLC algorithm	100	1PV module	4.08, 2.99	1000–600	0.1
^31^	IHHO algorithm	213.15	1PV module	13.51,33.33	1000–800	0.17
^32^	SSA algorithm	240	1PV module	19.22, 29.66	1000–500	0–2
^33^	AEO	120.7	4 parallel strings × 2 series-connected modules per string	15.15, 21.22	500–100	0–3
Proposed	The natural behaviour of pelican	349.59	3PV in series per string 2 parallel strings	4.32, 5.03	1000–600	0.2

Significant values are in underline

### POA based MPPT

To implement the POA for MPPT, the appropriate variables must be selected for the search process. Each pelican (agent) in the algorithm represents a candidate solution, and its performance is evaluated using an objective function. The goal of MPPT is to maximize power output, and the fitness function is typically defined based on the PV system’s P–V (Power–Voltage) or I–V (Current–Voltage) characteristics^34^. In this method, PV voltage values are considered as the candidate solutions (population) and the total number of these samples is denoted by n. The PV power at the MPP represents the value of the fitness function*.* The algorithm starts by initializing key parameters in the search space. Then, it determines voltage and current values as the initial population. For each sample, the corresponding power is calculated by multiplying voltage and current. This power is then used to assess the fitness of each candidate using Eq. (4), as shown in the flowchart.

**Fig. 5:**
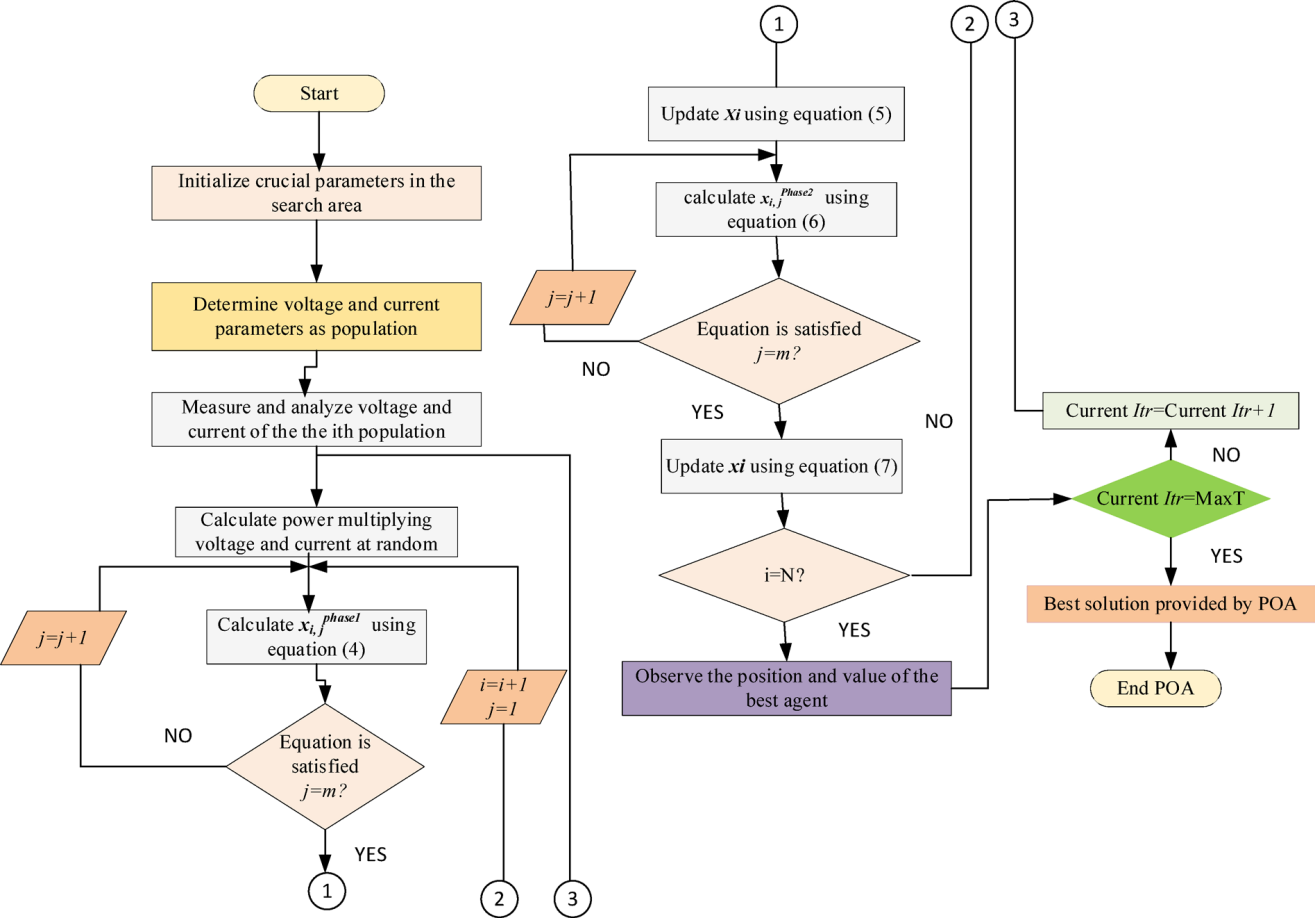
Flow chart of POA based MPPT.

The best candidate solution (i.e., the one producing the maximum power) is identified in the current population. The pelican’s position is then updated using Eq. (5), and a new position is calculated using Eq. (6). If the conditions are met, the position is further updated using Eq. (7). This process is repeated until the stopping criteria such as the maximum number of iterations is met. To improve search effectiveness, samples are distributed across the entire voltage spectrum, ensuring full exploration of the P–V curve. A larger number of samples (n) improves the algorithm’s accuracy and increases the likelihood of locating the global MPP. The POA balances exploration (searching new areas) and exploitation (refining known good areas) effectively. This balance allows the algorithm to avoid premature convergence and drift from the optimal solution. By continuously evaluating and updating positions around the best solutions (pelican positions), POA can efficiently converge toward the global maximum power point. According to the flowchart in Fig. 5, once the iteration limit is reached, the algorithm returns the best voltage and current combination that yields the maximum power, concluding the MPPT process. The pseudo code of the POA is shown in Appendix.

## Modelling of DC–DC converter

When the full load linked to the SPV structure receives consistent input, it performs well. As a result, it is critical to manage their input to meet their needs, because an SPV system’s output is in DC form. DC–DC converters are typically used to connect the system’s source and load to maintain the input at the desired level. Depending on the load, these types of converters either buck, boost, or conduct both buck-boost actions. Although basic buck-boost converters are less expensive, they suffer from electrical quantity ripple. To address this issue, strong LC filters are used, which raises their cost. They also have the problem of inverted output. All of these challenges are addressed by inventing new topologies that reduce voltage stress while increasing efficiency gain and are compatible with grid-connected devices. A modified high-power quality converter is used here to ensure the proper transfer of Energy from the source to the desired load. The DC–DC converter, incorporated with MPPT trackers, must have minimal input current fluctuation and balanced input and output power to ensure synchronization with the grid power. Compared to other topologies used with bipolar DC links, such as two or numerous switches to drive PWM for full or half-bridge topologies, this DC–DC converter utilizes only one switch; therefore, switching synchronization is unnecessary. The simulation runs for one second at a sampling interval of ten milliseconds.

### Switch ON period $(0 < t < DT_{s} )$

**Fig. 6:**
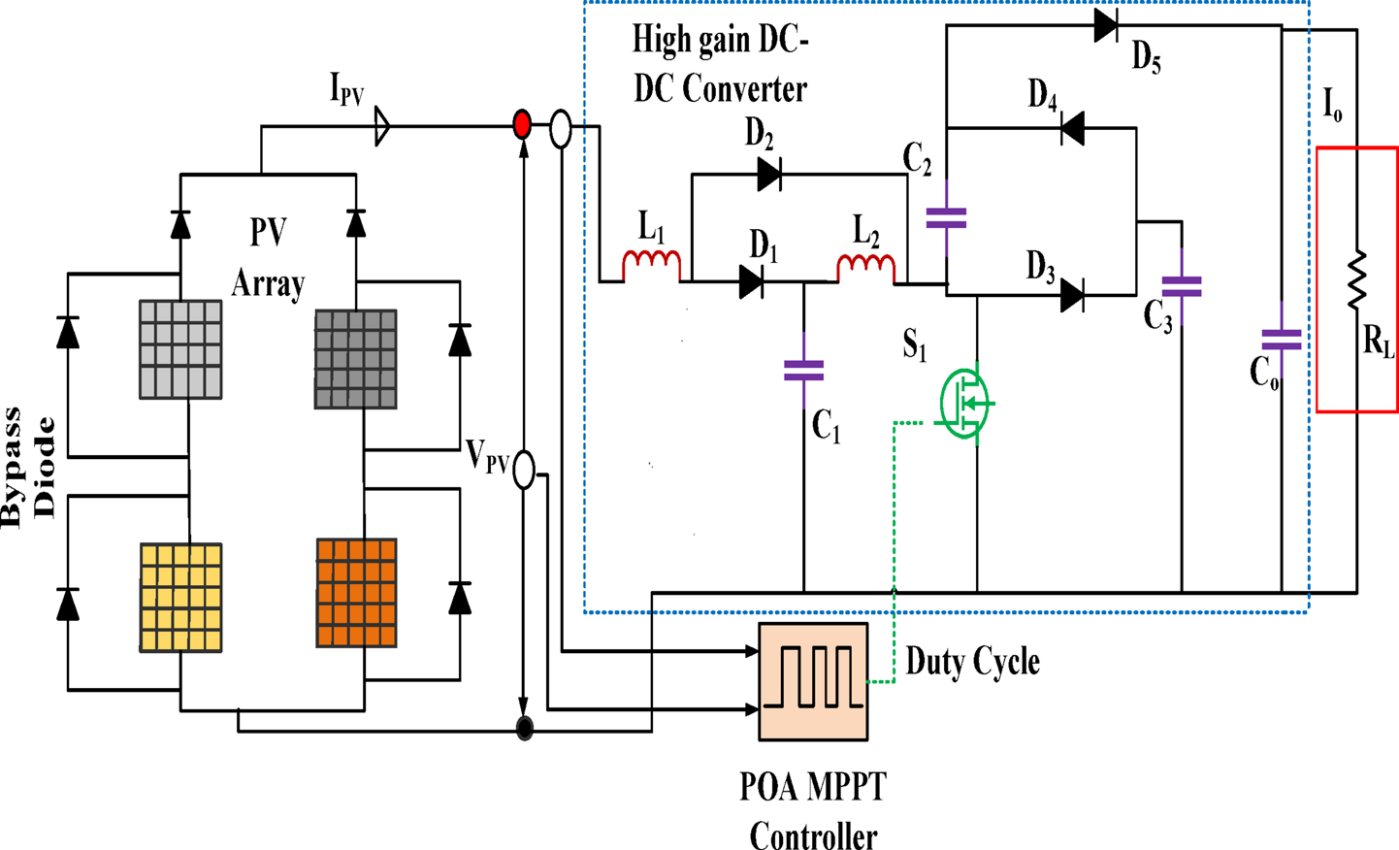
Standalone high gain converter.

Figure 6 depicts the schematic of the high-gain converter circuit. For the first operating mode, during this time, the primary switch was turned ON; hence, diodes D_2_ and D_4_ are carrying current. Here, L_1_ and D_2_ are where current from the source feed passes. In this case, the PV source provides energy to inductor L_1_, whereas C_1_ provides power to inductor L_2_. Consequently, the currents passing through the L_1_ and L_2_ increase proportionally.

**Fig. 7:**
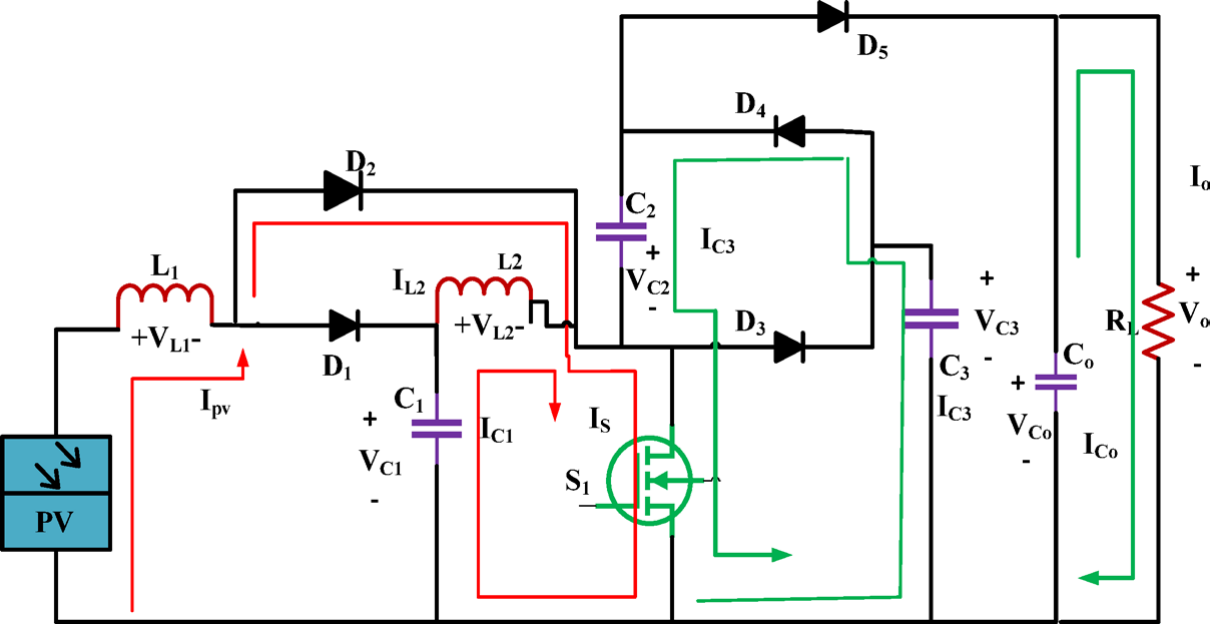
High gain ' ‘converter’s switch ON operating diagram.

Furthermore, C_3_ delivers Energy for recharging C_2_. In addition, the capacitor Co discharges, releasing its stored energy to run the load. Figure 7 shows the operating diagram.

### *Switch OFF period *$(DT_{s} < t < T_{s} )$

**Fig. 8:**
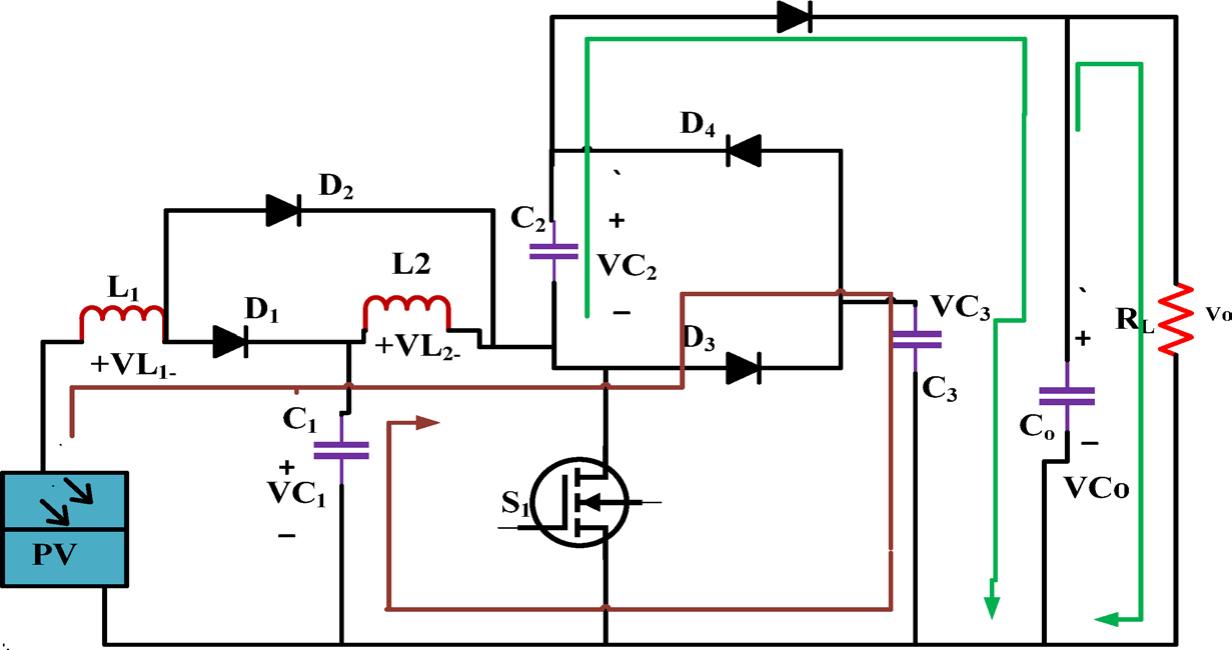
High gain ' ‘converter’s switch OFF operating diagram.

The diodes D_1_, D_3_, and D_5_ are active, whereas the others are not because the switch cannot conduct electricity. The corresponding circuit is shown in Fig. 8. As a result, L_1_ and L_2_ operate as a circuit that is coupled in series.The Energy kept in L_1_ and L_2_ is utilized to support loads through C_2_ and D_5_.

For inductive voltage volt second balance over one switching cycle is zero. For ON condition inductor voltage it can be written as

$$\begin{aligned} & \int\limits_{0}^{{T_{S} }} {V_{L1} } (t)dt = 0 \hfill \\ & V_{in} DT_{S} + (V_{in} - V_{C1} )((1 - D)T_{S} = 0 \hfill \\ & V_{in} DT_{S} + (V_{in} - V_{C1} )(T_{S} - DT_{S} ) = 0 \hfill \\ & V_{in} - V_{C1} + DV_{C1} = 0 \hfill \\ & V_{in} = V_{C1} (1 - D) \hfill \\ \end{aligned}$$

volt second balance For L_2_ can be written as,

$$\begin{aligned} & \int\limits_{0}^{{T_{S} }} {V_{L2} } (t)dt = 0 \hfill \\ & V_{C1} DT_{S} + (V_{C1} - V_{C3} )((1 - D)T_{S} = 0 \hfill \\ & V_{C1} DT_{S} + (V_{C1} - V_{C3} )(T_{S} - DT_{S} ) = 0 \hfill \\ & V_{C1} T_{S} - V_{C3} T_{S} (1 - D) = 0 \hfill \\ & V_{C1} T_{S} = V_{C3} T_{S} (1 - D) \hfill \\ & V_{C1} = V_{C3} (1 - D) \hfill \\ \end{aligned}$$

Rearranging the equition it can be written as

$$\begin{aligned} & V_{in} = V_{C1} (1 - D) \hfill \\ & V_{in} = (1 - D)V_{C3} (1 - D) \hfill \\ & V_{in} = (1 - D)^{2} V_{0} \hfill \\ & \frac{{V_{0} }}{{V_{in} }} = \frac{1}{{(1 - D)^{2} }} \hfill \\ \end{aligned}$$

The transfer function of the input and output voltage aiding voltage doubler part at the output can be written as.

8 $$\frac{{V_{0} }}{{V_{in} }} = 2\frac{1}{{(1 - D)^{2} }}$$

### Equation of Circuit Element

Typically, the level of ripple (approximately 5 and 10%) of the theoretical output current is used to determine the appropriate dimension and loss associated with the circuit element. The value for both inductors can be calculated using following equations

9 $$L_{1} = \frac{{V_{{_{PV} }} }}{{F_{s} \Delta IL_{1} }}D$$

10 $$L_{2} = \frac{{V_{{_{C1} }} }}{{F_{s} \Delta IL_{2} }}D$$

Similarly, taking the rated output voltage of the capacitor as the basis for calculation, a capacitor’s fluctuation in voltage value of [5–10%] is estimated.

11 $$\Delta Q = \frac{{I_{0} D}}{{F_{S} }}$$

12 $$\Delta V_{C} = \frac{\Delta Q}{C}$$

13 $$C = \frac{{I_{0} D}}{{\Delta V_{C} F_{S} }}$$

## Result and discussion

To assess the potential of POA through Simulink, four widely used MPPT techniques—P&O, PSO, CS, and GWO are used to compare their performance. The following test is applied to all five algorithms. Temperature and irradiance change continuously, (2) abrupt irradiance step changes, (3) quick changes in steps in both temperature and Irradiance, and (4) the capacity to manage partial shade conditions. An explanation of each performance parameter, contrasted with other existing optimization algorithms, is stated in the upcoming section. Photovoltaic panel simulation specifications are tabulated in Tables 1 and 2, respectively. To operate a high-gain converter, the value of both inductors used for simulation is 200 µH, whereas the capacitor values are 800 to 1000 µF. The output is measured across a 200Ω resistor.

### Fixed irradiance and temperature analysis

**Fig. 9:**
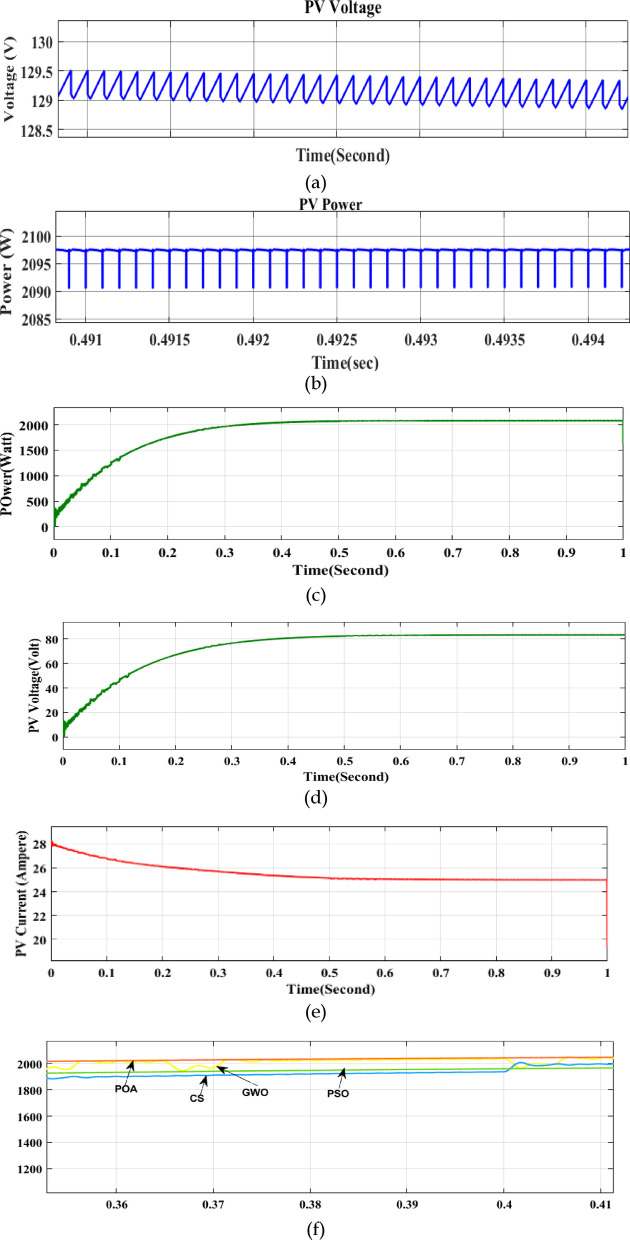
Zoom view of (**a**) PV voltage (**b**) Power (**c**) Transient and steady-state PV power, (**d**) voltage, (**e**) current (**f**) Comparison of different algorithms (**g**) Duty cycle, and (**h**) applied PWM to drive the switch.

The purpose of this test is to ascertain the starting speed. The modules listed in Table 1 are used to construct the PV array. Peak power 2095 W is the estimateat STC. Figure 8a and b displays a closer view of the voltage and power responses that resulted. The MPP gets tracked in 0.2 s, as shown in Fig. 9a. This is quick enough to keep up with the actual variation in the atmosphere since it takes a few seconds or more to adjust for variations in temperature and brightness. One crucial aspect of POA is that, under steady-state circumstances, the operating point stays fixed at MPP, meaning that oscillation is non-existent.

In contrast, fluctuations around the MPP are common in P&O (and other typical approaches). Figure 9d and e show the PV voltage and current scenario. A comparison of POA with four different optimization techniques is shown in Fig. 9f. The PV output indicates that the Proposed POA MPPT performs better than others. Figure 9g shows the stabilization of the duty cycle after 0.2 s. The PWM signal given to the gate of the switch is displayed in Fig. 9h.

### Step adjustment to the irradiance at a fixed temperature

**Fig. 10:**
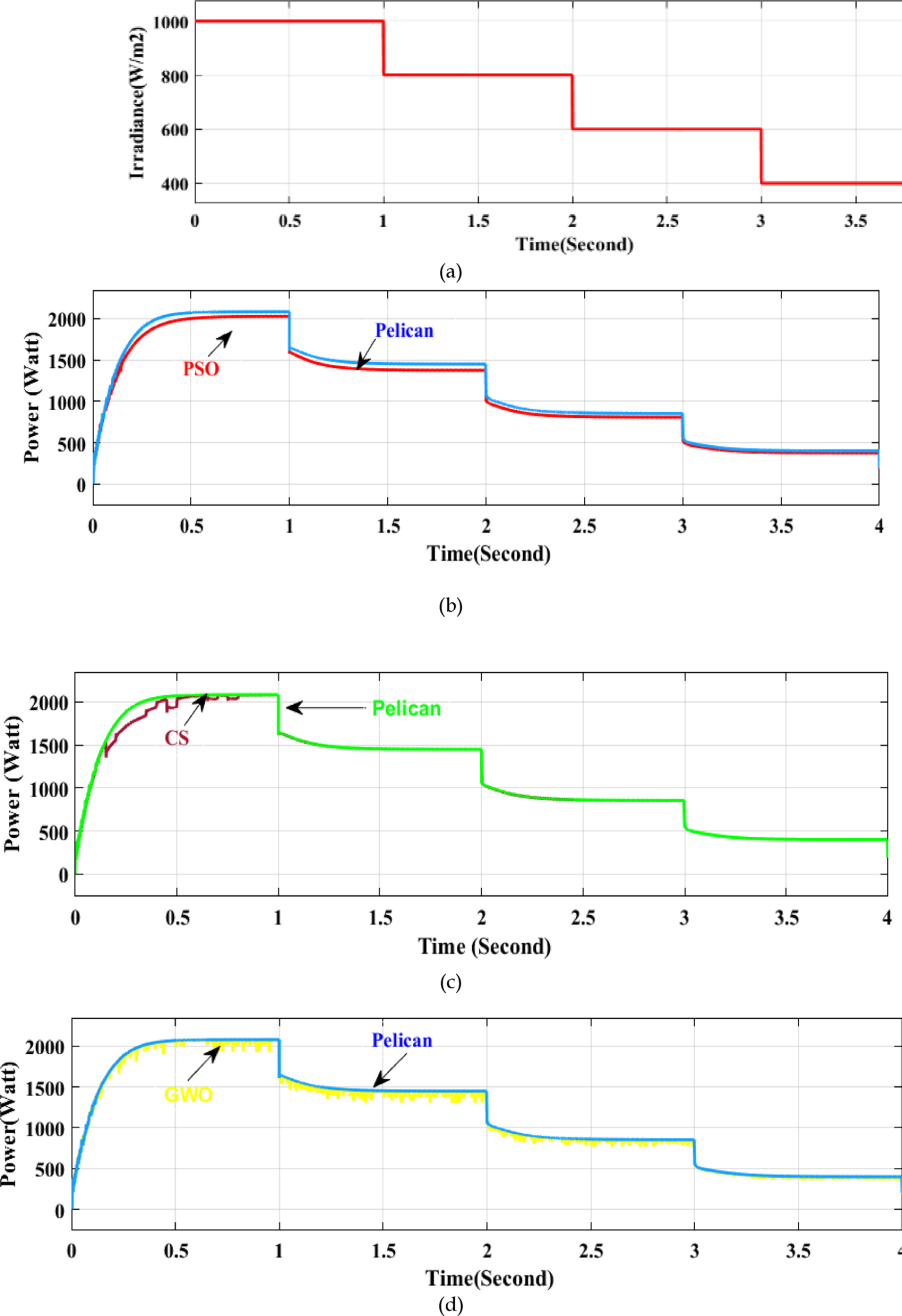
(**a**) Non-uniform irradiance test: Irradiance level, (**b**) Power comparison of PSO and Pelican at different Irradiance, (**c**) Power comparison of CS and Pelican at different Irradiance, (**d**) Power comparison of GWO and Pelican at different Irradiance, (**e**) Power comparison of P&O and pelican at fixed IIrradiance.

Step changes in radiation occur when a cloud moves quickly across a photovoltaic array. A series of irradiance levels are applied to the array to assess the algorithm’s performance under these circumstances, and the step adjustment is implemented once every second. The experiment is conducted at a set temperature of 25 °C. Figure 10 displays the step reaction for each optimization algorithm. A predetermined step size of 5 V is chosen for the P&O to prevent significant oscillation around the MPP. Moreover, P&O fluctuates (around the MPP) with an average ripple, even at modest step sizes, as shown in Fig. 10e. However, the loss is essentially nil since POA consistently adheres to the MPP. Figure 10b, c, and d compare the capabilities of CS, PSO, and GWO to track MPP at varying irradiance. PSO needs 600 ms for the preliminary MPP tracking. When settling into a new MPP value, PSO takes about 120 ms, while CS takes 100 ms. GWO oscillates throughout the MPP range, responding to each irradiance level. The greater step size and the perfect balance between exploration and exploitation are most likely the cause of the faster reaction of POA. Furthermore, since the algorithm must run more tests before converging to MPP, PSO displays longer swings in the transitory state. However, in stable conditions, both approaches precisely follow the MPP.

### Steps shift in irradiance and temperature

This simulation aims to ascertain how the simultaneous step adjustments to temperature and IIrradiance will affect the MPPT algorithms. Such a sharp shift in solar radiation is not uncommon, especially in tropical nations before and during the monsoon season when thick clouds quickly obscure the sun. Although it is unlikely that temperature will fluctuate rapidly, step fluctuation is included to evaluate the algorithm’s performance in harsh environmental circumstances. The search starts when POA scatters its samples throughout the P–V curve at the step function’s edge. The power oscillation is more noticeable because the temperature step variation is also considered. As a result, the POA samples are spread throughout a wider voltage span. Acknowledging that the power fluctuations are limited to a few hundred milliseconds from the step’s edges is essential. Comparing these transient behaviors to the constant volatility of P&O at a steady state, the PV system’s overall yield may not be significantly impacted. As shown from the above figure, all mentioned methods precisely track the MPP in a constant state. Nevertheless, POA outperforms PSO, CS, GWO, and P&O in terms of transitory performance.

### Simulink study

PV system (2S-2P size) is considered for MATLAB Simulink study during shading conditions such as non-uniform irradiance levels. In PV system, total four numbers of modules are used and arranges in series –parallel connections. 5W commercial PV module specifications (I_m_ = 0.52A, I_SC_ = 0.55A, V_m_ = 9.62 V, P_m_ = 5W, Model- SFTI18P5, Manuf.- Solar Universe India) are considered for Simulink and experimental validation studies.

**Fig. 11:**
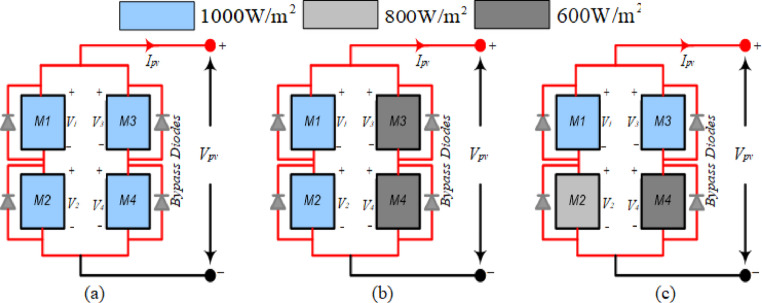
PV system (2S-2P) with (**a**) Case-I (**b**) Case-II (**c**) Case-III.

Three scenarios (I–III) depending on irradiation levels are examined for the performance evaluation of the PV system, conducted using a MATLAB/Simulink research, as illustrated in Fig. 11a–c. In Case I, the PV modules M1, M2, M3, and M4 exhibit identical irradiance values of 1000 W/m^2^. In Case II, PV modules M1 and M2 are subjected to irradiation intensities of 1000 W/m^2^. However, PV modules M3 and M4 are situated in the second strings and encountered shadowing situations at irradiance levels of 600 W/m^2^. Additionally, Case-III examined the non-uniform irradiance levels of 100 W/m^2^, 800 W/m^2^, and 600 W/m^2^ for all the PV modules (M1, M2, M3, M4), as seen in Fig. 11c.

**Fig. 12:**
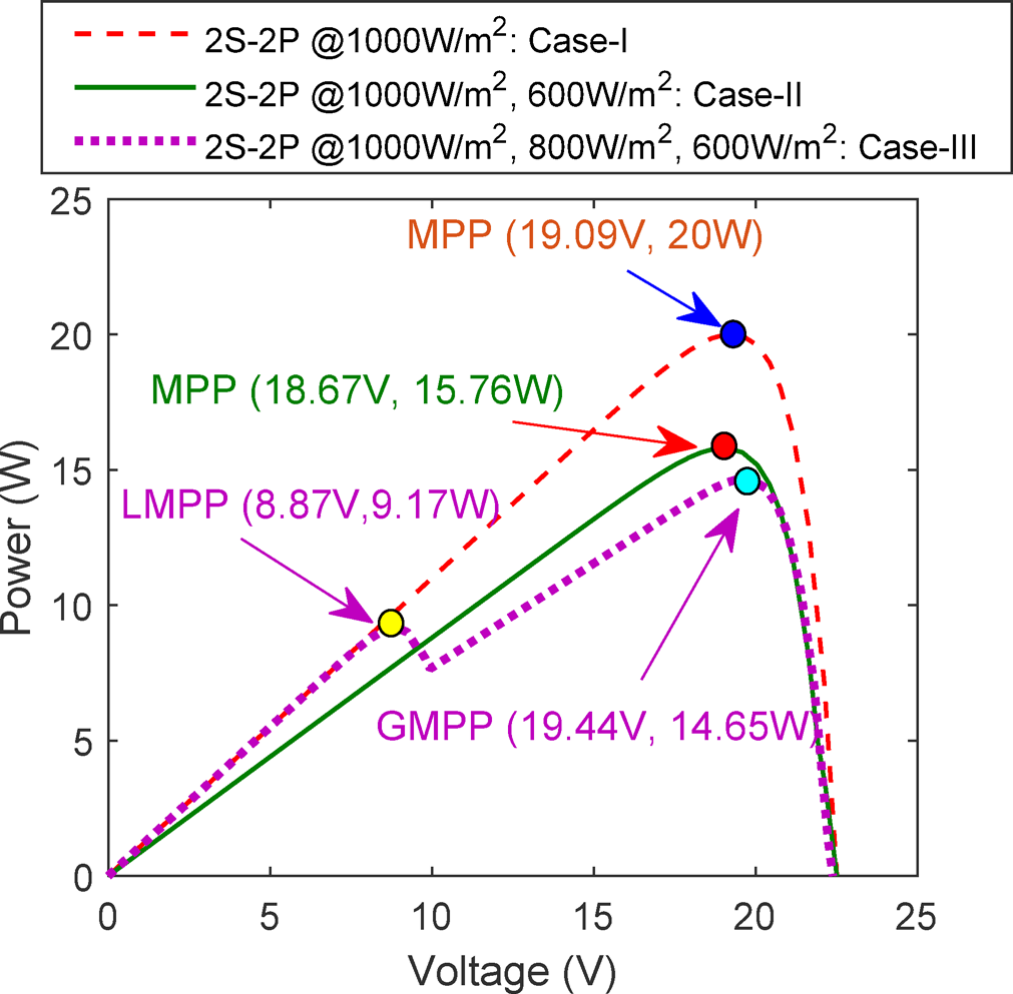
Existence of MPP, LMPP & GMPP on P–V curves due to non-uniform irradiance levels for 2S-2P arrangement.

The performance parameters for MPP for case I and II are 19.09 V, 20W and 18.67 V, 15.76W, respectively. Furthermore, the effects of partial shading are evident by the presence of several maximum power points; specifically, the local maximum power point (LMPP) and global maximum power point (GMPP) for case-III are recorded at 8.87 V, 9.17W and 19.44 V, 14.65W, respectively, due to non-uniform irradiation levels. Figure 12 illustrates the positions of MPP, LMPP, and GMPP according to the analysed solar irradiation in Cases I-III using the P–V curves.

### Experimental verification

**Fig. 13:**
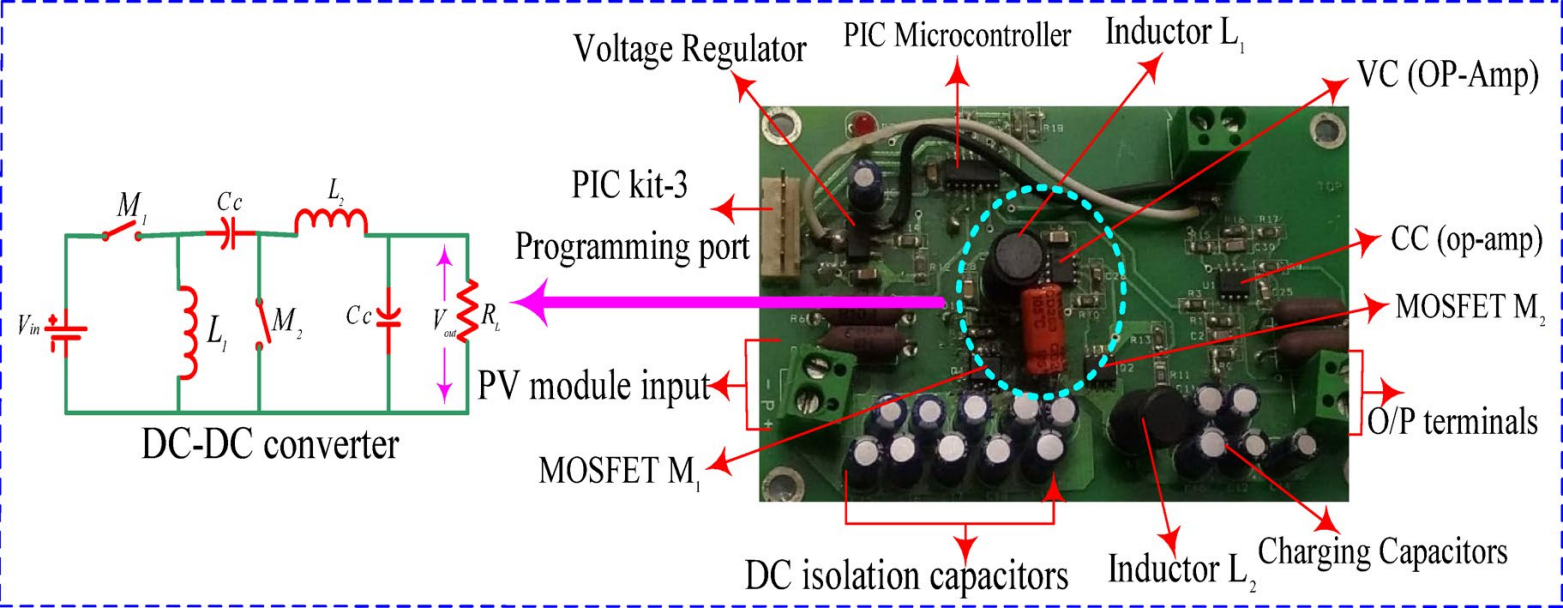
Schematic diagram of DC–DC converter and MPPT development.

**Fig. 14:**
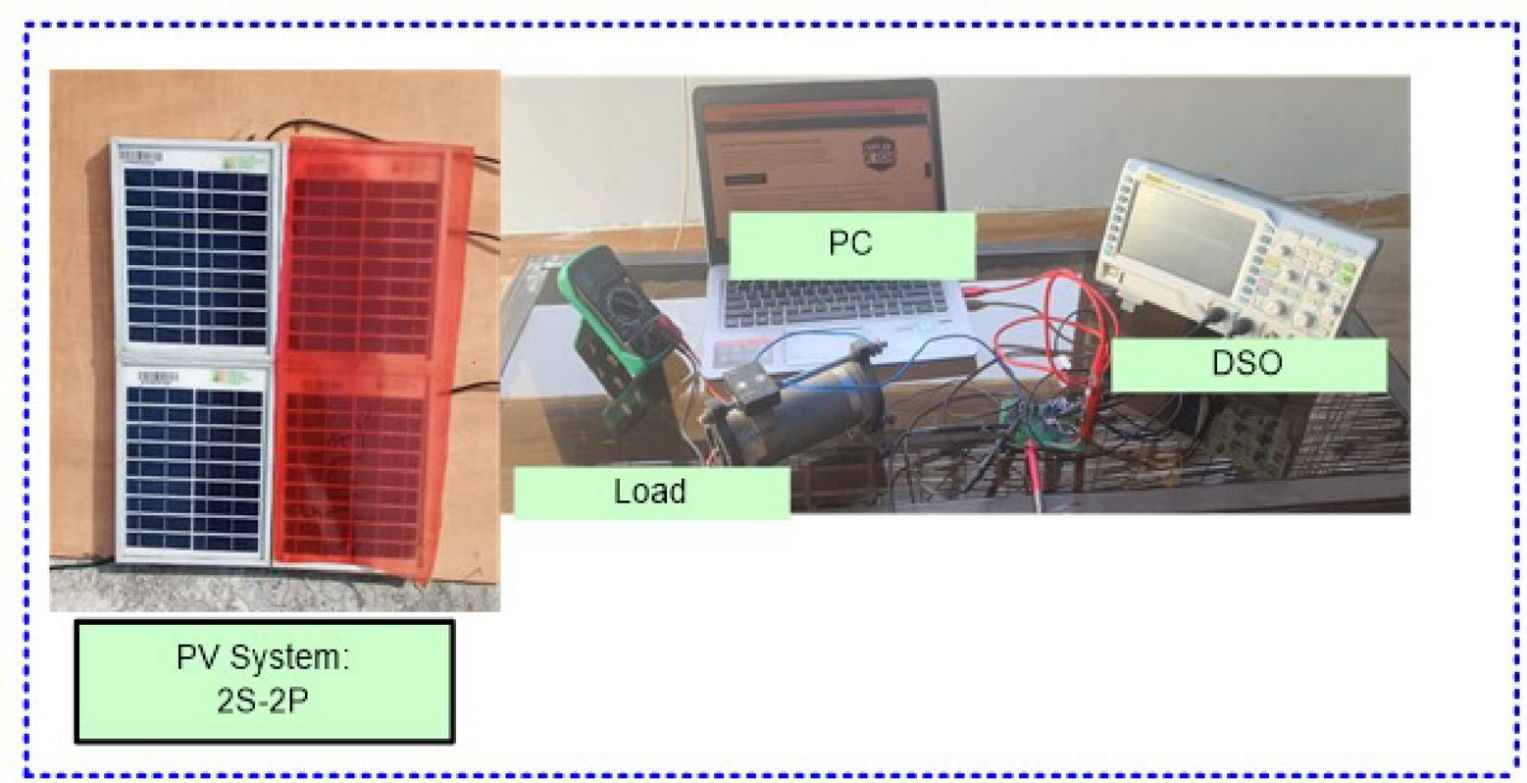
Experimental setup of the proposed algorithm.

The lab develops the physical model to verify the operation under various weather conditions. The outcomes of the POA were in contrast to that of the PSO approach. MPPT code was sent from the host computer to the pic 16 microcontrollers via Pic Kit 3. The layout utilizes a standalone prototype SPV. Four solar panels (B07XQ8KTF5) are connected in a 2 × 2 SP configuration. An embedded PIC 16F family-series microprocessor on the PCB board regulates the converters’ duty cycles. The SPV system contains a resistive load to measure the MPPT approach’s efficacy for PSCs. The algorithms have been evaluated under various load circumstances utilizing a POT, an adjustable load for the SPV system. The DC Crompton voltmeter approach detects real-time current by measuring the potential drop over a 1 Ω resistance in series with the load. Thin plastic sheets form PSCs on the SPV array based on irregular irradiance intensities. DSO is additionally employed to record transient events for electrical performance monitoring. Figures 13 and 14 illustrates the schematic diagram of DC–DC converter –MPPT and experimental setup as follows,

In the circuit development of MPPT, the converter core is developed through two n- channel MOSFETs devices $\left( {{\text{M}}_{{1}} {\text{,M}}_{{2}} } \right)$ and a coupling capacitor $\left( {{\text{CC}}} \right)$**.** In addition, the buffer capacitors are required at input and output ports for smooth operation and efficiency of the device. The most important component such as PIC micro-controller IC [52] is used to regulate both the MOSFETs devices. Furthermore, MOSFET device-1 $\left( {{\text{M}}_{{1}} } \right)$ is kept as in ‘ON’ state, inductor-1 ${\mathbf{L}}_{1}$ $\left( {{\text{L}}_{{1}} } \right)$ stores the energy and provides to output side through the ${\text{CC}}$ along with inductor-2 $\left( {{\text{L}}_{{2}} } \right)$**.**

As a next stage, MOSFET device-2 $\left( {{\text{M}}_{{2}} } \right)$ is kept as in ‘OFF’ state, and ${\mathbf{C}}_{\mathbf{C}}$ ${\text{CC}}$ is discharged through the inductor-1. Additional capacitor is called as charging capacitor is attached at output side to deliver the current to load continuously. Overall, process is on-going to control the duty cycle with the effective switching frequency rises from 0 to 550 kHz (90% duty cycle).

Due to the lower duty cycle at lower input voltages and higher input voltages, a higher duty cycle provides more value. All the supporting components used in the MPPT development and associated with experimental set up are reported in Table 4 as,

**Table 4:** Performance comparison of four metaheuristic algorithms.

MPPT algorithm	Tracking time	Tracking efficiency (%)	Fluctuation at transient	Steady-state oscillation (%)	Work on partial shading
P&O^34^	0.25	98	High	0.2	N
PSO^35^	0.23	97.8	Moderate	0.005	Y
CS^18^	0.22	97.6	Low	0.00008	Y
GWO^17^	0.24	97.7	High	0.004	Y
POA[proposed]	0.20	99	Low	0.00006	Y

**Fig. 15:**
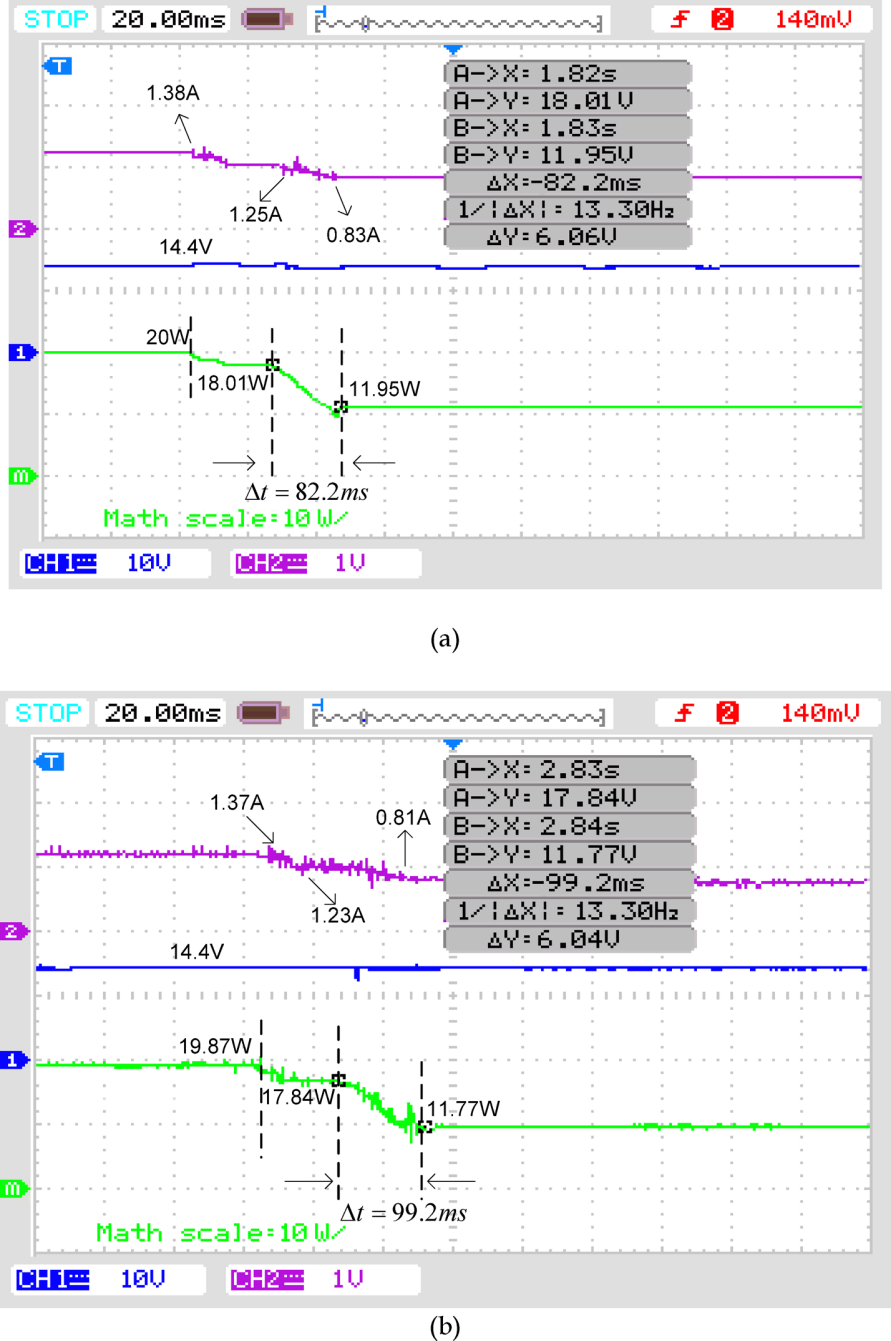
Performance Comparison of (**a**) POA and (**b**) PSO.

The performance of the SPV system is evaluated by following the voltage, current, and power output forms under the required conditions using the specified DC converter. Channel 1 of the DSO displays the SPV unit’s voltage curve, while channel 2 displays the corresponding current curve. A numerical multiplication function is used to evaluate the system’s power output graph. Each section on the DSO monitor indicates 10 V of voltage, 1 A of current, and 10 W of power. The transient response screen shows real-time electrical output data, including values of power on the Y-axis as Irradiance varies. X represents the time it takes to move from the preceding value to the next one. Figure 15a demonstrates the POA execution, while Fig. 15b shows the PSO outcomes. Figure 15a shows the corresponding current and power graphs as Irradiance quickly reduces from 1000 to 800 W/m^2^ and then from 800 to 600 W/m^2^. When, the Irradiance reduces from 1000 to 800 W/m^2^, the current decreases from 1.38A to 1.25A. Moreover, the Irradiance drops from 800 to 600 W/m^2^ current further drops to 0. 83A.The voltage stayed constant at 14.4 V. The observation of the power is found correspondingly as 20W, 18.01W, and 11.95W respectively. Figure 15b illustrates the associated current and power graphs as irradiance rapidly decreases from 1000 to 800 W/m^2^, and subsequently from 800 to 600 W/m^2^. As the irradiance diminishes from 1000 to 800 W/m^2^, the current declines from 1.37 A to 1.23 A. Furthermore, the irradiance decreases from 800 to 600 W/m^2^, resulting in a current reduction of 0.81 A. The voltage remained stable at 14.4 V. The observed power measurements are 19.87 W, 17.84 W, and 11.77 W, respectively.

It has also been demonstrated that the DC–DC converter sets down in the shortest time, 82.2 ms, while operating with POA, whereas PSO algorithm takes longer, 99.2 ms, with similar shading scenarios. In addition, the results reveal that the circuit sets to maximum power while operating with POA but to low power when using another algorithm.

### MPP tracking

Table 5 comes up with a summary of the tracking capacity for ease of use. Despite being ranges rather than absolutes, the numbers can be regarded as reliable indicators of how well different techniques perform in comparison. Numerous factors, including the power level, sample location, and random number uncertainty, contribute to the variations. POA seems the fastest for the first power rise from zero to stable. The higher step sizes as a result of the Lévy flight can be used to explain the rapid convergence of CS. P&O operates at a speed that is comparable to POA. This is predicted as the approach only needs to progressively rise with a set step size from zero to MPP. Step size can be raised for faster convergence, although doing so will cause steady-state oscillation. The GWO is the slowest; it takes 300–600 ms to attain a stable state. This is because the vector summing between its local and global best particle results in a reduced step size. It typically takes only 50 ms for MPP retracting to adjust to a new MPP. This is so because P&O solely relies on the PV curve’s gradient, making the ascent in the direction of MPP considerably simpler. However, PSO, CS, and GWO are search-based methodologies. The algorithms will scatter their particles throughout the voltage span for each significant change in temperature or Irradiance; only then can the search be started. Moreover, the tracking algorithm is quite susceptible to the random numbers that are created. In terms of transitory performance, it’s interesting to observe that, in contrast to existing optimization, POA shows less power fluctuation even if it’s quicker.

**Table 5:** DC–DC converter MPPT components and specifications.

Components	Specifications
Capacitor	220 pf–48 µf
Resistances	0.1 k–200 kΩ
Microcontroller	PIC 16F15325
Regulator	SI4154
Scaling factor (N)	2
Inductor (L)	30–88.4 mH
Capacitor (C)	40 F
Resistance	1 Ω

### Fluctuation at steady state

The primary benefit of metaheuristic algorithms is their lack of MPP oscillations, which results in nearly minimal energy loss at a steady state. Continuous oscillation results in significant power loss for P&O, especially for big PV installations. Even if CS, PSO, GWO, and POA show power variation during transient, this behavior may not significantly impact the PV system’s total output.

### Algorithm complexity

The step size is the sole tuning parameter that the P&O needs. The algorithm is simple to understand and apply. The three parameters for PSO that require tuning are w, c1, and c2. Experience has shown that a thorough process of trial and error is necessary to identify the ideal mix for maximum performance. However, just two settings need to be adjusted for POA. Programming requires less work overall.

### Handling partial shading

To compute the proportion of partial shading that impacts a PV panel, considering the sun’s radiation and temperature, we need to examine how much the partially shaded region reduces the overall power production in comparison to full sun. Power obtained from the partial shading impact can be calculated as

$${\text{Partial}}\;{\text{Shading}}\;{\text{Impact(\%)= }}\left( {\frac{{{\text{P}}_{STC} - {\text{P}}_{Shaded} }}{{{\text{P}}_{STC} }}} \right) \times 100$$

Using above mention formula Percentage impact for partial shading can be calculated. For standard testing condition irradiance is 1000W/m^2^ and temperature is 25 °C. Due to shading impact the percentage become 20% for irradiance of 800 w/m2 and 40% for irradiance of 1000W/m^2^. This suggested method is very can examine the P–V curve and find the GMPP, even when there are many local maxima owing to shading.

P&O cannot manage the partial shading scenario at all. This can be important in many cases, especially when installing integrated PV systems in locations with high population density. PSO, CS, GWO, and POA are adept at handling partial shading. However, considering additional factors like tracking ability, transient power fluctuation, and convergence speed, POA can be deemed a superior choice.

**Fig. 16:**
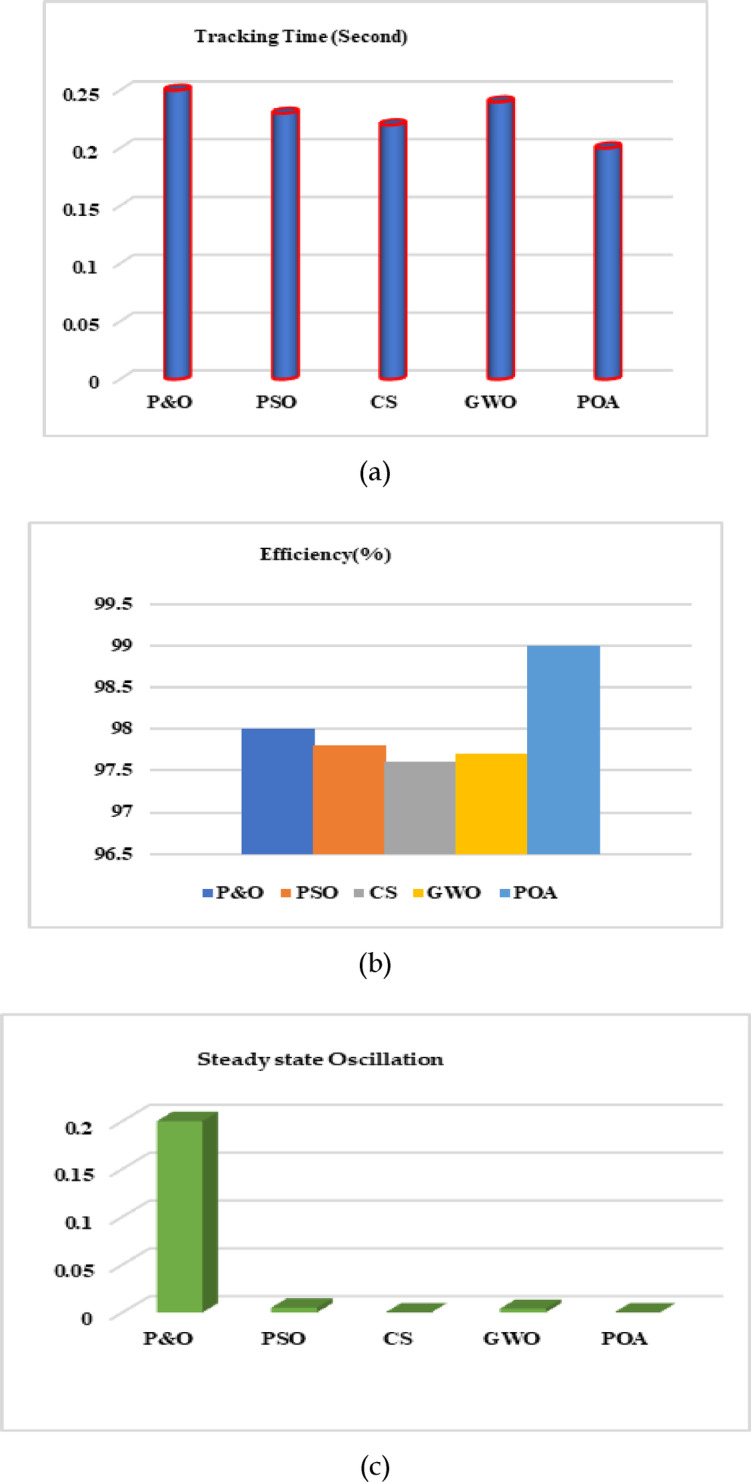
Bar diagram representation of different metaheuristic algorithms based on (**a**) tracking time (**b**) tracking efficiency (**c**) steady-state oscillation.

The performance of each algorithm highlighting tracking time, tracking speed, and steady-state oscillation is shown separately in Fig. 16 using a bar diagram for better comprehension.

### Evaluation of the converter performance

**Fig. 17:**
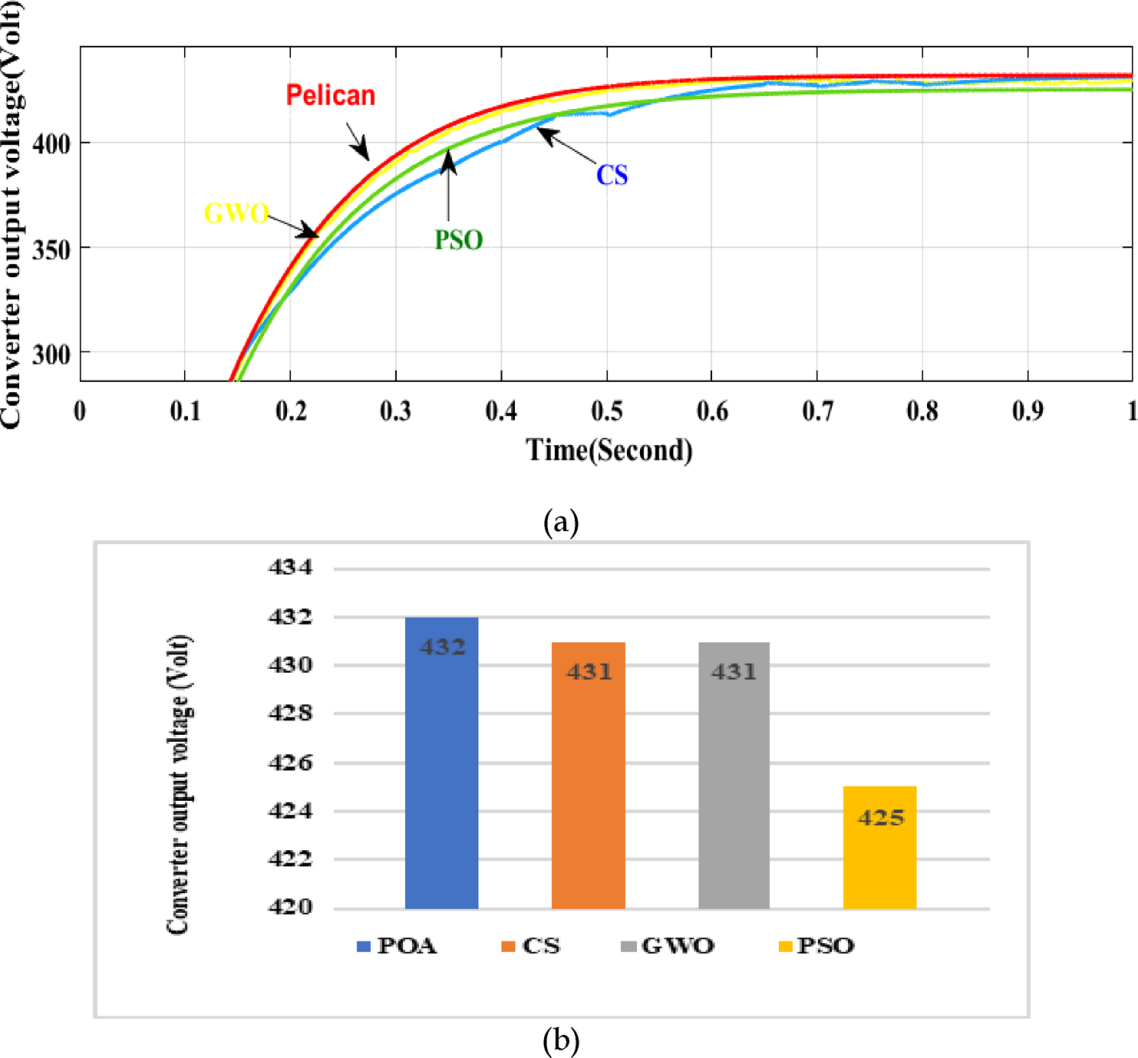
(**a**) Output of the converter employing different optimization techniques (**a**) Simulation output (**b**) comparison in bar diagram.

This high gain converter has been adopted with a PV source utilizing four metaheuristic algorithms. The simulation output in terms of voltage gain is expressed in Fig. 17. From the graphical analysis, and bar representation it is pretty understandable that POA MPPT demonstrates better results than others with regard to gain and fluctuation in voltage.

### Converter performance in terms of gain and efficiency

High-gain DC–DC converters play a crucial role in enhancing the effectiveness of PV-fed maximum power point tracking (MPPT) systems. Their main advantage is that they are able to effectively boost low PV voltages, particularly under partial shade or low Irradiance, to the desired DC bus level without requiring numerous conversion stages.

**Fig. 18:**
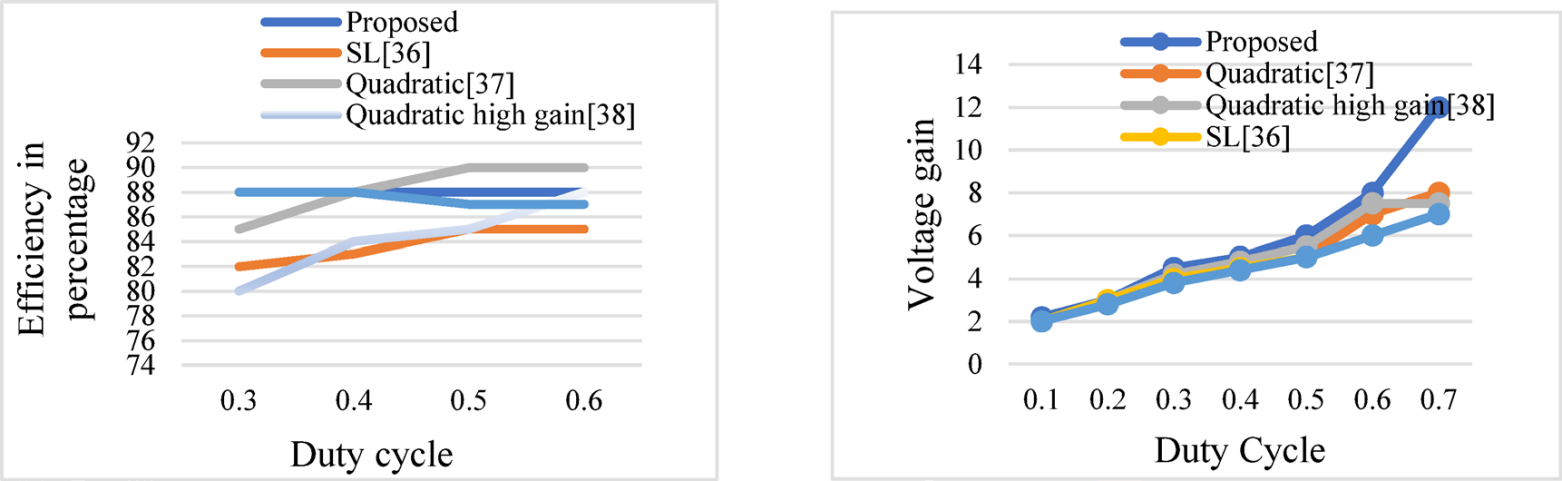
(**a**) Efficiency and (**b**) Voltage gain comparison.

These converters increase the MPPT operating range, enabling the system to follow the GMPP irrespective of adverse conditions. They also decrease the requirement for long series PV panel connections, which reduces mismatch losses and simplifies system design^36–38^. Performance comparison of various high-gain topologies concerning efficiency and voltage gain is given in Fig. 18a, b.

## Conclusion

This study proposes a new MPPT approach based on POA to obtain optimum power from a PV source. This unique approach occupies a smaller memory in the PIC microcontroller’s ROM and takes a shorter period to track, thus rendering it less complicated. The proposed approach outperforms previous tactics regarding tracking efficacy, tracking duration, and robustness based on findings and quantitative and analytical data processing. The suggested MPPT strategy is experimentally examined using the widely recognized PSO technique. In addition, the four commonly used MPPT algorithms, P&O and PSO, GWO, and CS, are simulated in MATLAB to benchmark the performance of POA. As per the tabulated outcome shown in Table 4, The POA outperforms other algorithms regarding tracking time (around 20% faster than P&O), tracking efficiency (approximately 1.02% more than P&O), and steady-state oscillation (around 25% less than the CS algorithm). It is also demonstrated that the POA can track global MPP when partially shaded. Moreover, the proposed MPPT contributes to solar power tracing by optimizing solar panel execution, adapting to varying conditions, performing a search for GMPP, converging fast, and maintaining robust functionality in the face of ambiguity. A newly developed high-gain DC–DC converter is used as an interfacing circuit between the PV source and load. In addition, great attention is paid to selecting the values of the circuit components (switches, inductors, and capacitors) employed in the simulation to ensure they match the actual hardware scenario. Compared to other existing algorithms, POA performs well in terms of converter voltage gain. The PV community, comprising researchers and practitioners, is anticipated to be highly interested in this work.

## Figures and Tables

**Table 6:**
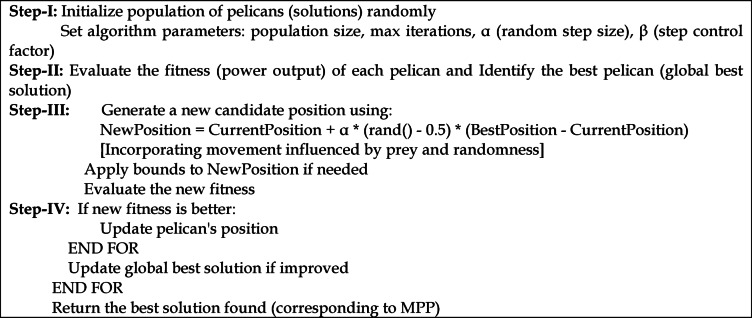
Pseudocode of the POA algorithm.

**Table 8:**
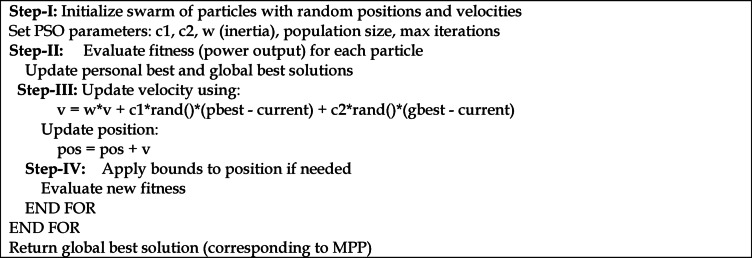
Pseudocode of the PSO algorithm.

## Data Availability

The datasets used and/or analysed during the current study are available from the corresponding author upon reasonable request.

## Appendix

Pseudocodes and Parameters settings of the POA and PSO algorithms (Tables 6, 7, 8 and 9).

**Table 7:** Parameters settings of POA algorithm.

Parameters	Values
Population size	30
Max iterations	100–200
α (step size)	0.5
β (learning factor)	0.3
Search space	PV voltage range (e.g., 0–19.8 V)

**Table 9:** Parameters settings of PSO algorithm.

Parameters	Values
Population size	20–30
Max iterations	100–200
c1 (cognitive)	2
c2 (social)	2
w (inertia weight)	0.4–0.9
Search space	PV voltage range (e.g., 0–19.8 V)
